# The Medical Record

**Published:** 1872-12

**Authors:** 


The Medical Record.—This excellent journal completes its seventh volume with the present year. Although it has been published but a short time, it has taken rank among the foremost of medical periodical publications, and has received the support of some of the most prominent members of the profession. We have ourselves often taken the opportunity of making selections from its pages, and our readers will bear us witness of their general worth and excellence. We congratulate the editor and publishers of The Record on their success thus far and trust that the future has much good in store for them.

